# A Web-Based Graphical Food Frequency Assessment System: Design, Development and Usability Metrics

**DOI:** 10.2196/humanfactors.7287

**Published:** 2017-05-08

**Authors:** Rodrigo Zenun Franco, Balqees Alawadhi, Rosalind Fallaize, Julie A Lovegrove, Faustina Hwang

**Affiliations:** ^1^ Biomedical Engineering School of Biological Sciences University of Reading Reading United Kingdom; ^2^ Hugh Sinclair Unit of Human Nutrition and Institute for Cardiovascular and Metabolic Research Department of Food and Nutritional Sciences University of Reading Reading United Kingdom

**Keywords:** nutrition assessment, FFQ, food frequency questionnaire, personalized nutrition, nutrition informatics, dietary intake, usability, SUS

## Abstract

**Background:**

Food frequency questionnaires (FFQs) are well established in the nutrition field, but there remain important questions around how to develop online tools in a way that can facilitate wider uptake. Also, FFQ user acceptance and evaluation have not been investigated extensively.

**Objective:**

This paper presents a Web-based graphical food frequency assessment system that addresses challenges of reproducibility, scalability, mobile friendliness, security, and usability and also presents the utilization metrics and user feedback from a deployment study.

**Methods:**

The application design employs a single-page application Web architecture with back-end services (database, authentication, and authorization) provided by Google Firebase’s free plan. Its design and responsiveness take advantage of the Bootstrap framework. The FFQ was deployed in Kuwait as part of the EatWellQ8 study during 2016. The EatWellQ8 FFQ contains 146 food items (including drinks). Participants were recruited in Kuwait without financial incentive. Completion time was based on browser timestamps and usability was measured using the System Usability Scale (SUS), scoring between 0 and 100. Products with a SUS higher than 70 are considered to be good.

**Results:**

A total of 235 participants created accounts in the system, and 163 completed the FFQ. Of those 163 participants, 142 reported their gender (93 female, 49 male) and 144 reported their date of birth (mean age of 35 years, range from 18-65 years). The mean completion time for all FFQs (n=163), excluding periods of interruption, was 14.2 minutes (95% CI 13.3-15.1 minutes). Female participants (n=93) completed in 14.1 minutes (95% CI 12.9-15.3 minutes) and male participants (n=49) completed in 14.3 minutes (95% CI 12.6-15.9 minutes). Participants using laptops or desktops (n=69) completed the FFQ in an average of 13.9 minutes (95% CI 12.6-15.1 minutes) and participants using smartphones or tablets (n=91) completed in an average of 14.5 minutes (95% CI 13.2-15.8 minutes). The median SUS score (n=141) was 75.0 (interquartile range [IQR] 12.5), and 84% of the participants who completed the SUS classified the system either “good” (n=50) or “excellent” (n=69). Considering only participants using smartphones or tablets (n=80), the median score was 72.5 (IQR 12.5), slightly below the SUS median for desktops and laptops (n=58), which was 75.0 (IQR 12.5). No significant differences were found between genders or age groups (below and above the median) for the SUS or completion time.

**Conclusions:**

Taking into account all the requirements, the deployment used professional cloud computing at no cost, and the resulting system had good user acceptance. The results for smartphones/tablets were comparable with desktops/laptops. This work has potential to promote wider uptake of online tools that can assess dietary intake at scale.

## Introduction

Food frequency questionnaires (FFQs) are a commonly used tool for dietary assessment, and paper-based FFQs have been used for decades in the field of human nutrition [1-2]. An FFQ consists of a list of food and drink items, and for each item, an individual indicates their typical consumption frequency and portion size, based on their dietary intake for a given reference period (eg, the past month). The list of foods is based on the most frequent foods in the region and typically has around 100 items. Consumption frequencies are normally indicated using categories described in text (eg, 1 per day). Portion sizes can be indicated by selecting text-based categories (eg, small, medium, or large) or by selecting the closest match from a selection of portion-size photographs of actual foods [3]. There have been studies published on the validity of FFQs in different countries, in both paper-based and digital versions [4-13]. FFQs are frequently used in epidemiological (ie, population) studies as they are inexpensive to process, can be self-administered, and are relatively quick for participants to complete [14-15]. However, they are also prone to reporting bias; the consumption of healthy foods has been overestimated using this method [16-17].

FFQs have traditionally been delivered using a pen-and-paper format, but there is a burden associated with this format for study participants, health professionals, and investigators. The digitalization of nutrition assessment methods has excellent potential to save time and resources, is preferred by participants [18], and is more suitable for large-scale studies. Other online dietary assessment methods such as the 24-hour recall [19-21] claim better accuracy than FFQs. However, the motivations for investigating online FFQs include that they are easier to replicate technically than these other methods, which often require a much larger food database and more complex technologies such as text search functionality; FFQs may also be more suitable for certain applications including online personalized nutrition interventions [22]. Although some Web-based FFQs have been developed in recent years, they have not been used widely in this format as yet, and there are few published results in terms of user acceptability of online FFQs.

In order to facilitate the dissemination of online FFQs, it is important that the scientific and public health communities have open and free access, not only to the final results of validation studies but also to the design, architecture, development, and deployment of scalable, replicable, and secure tools. Furthermore, interdisciplinary collaboration and shared understanding between the health and technical communities is important for furthering research in this field, and as such, it is appropriate that studies also report their work from the perspectives of multiple disciplines. Therefore, this paper presents both the technical design of a Web-based graphical food frequency assessment system and results from user testing, with an aim of making a contribution to the wider uptake of digital FFQs.

The online FFQ described in this paper was designed and developed for the Eat Well Kuwait project (EatWellQ8, www.eatwellq8.org), which aims ultimately to investigate whether Web-based personalized nutrition (based on dietary intake and anthropometrics) is as effective as face-to-face communication of personalized nutrition in Kuwait. The project is a collaboration between the University of Reading and the Dasman Diabetes Institute in Kuwait City [23]. The first stage of this project focused on the design and development of the Web-based FFQ, and a validation study is currently under way to compare the online FFQ with the current paper version of a Kuwaiti FFQ and a 4-day weighed food record.

### Objectives

#### Overview

This paper aims to make a contribution to the wider uptake of digital FFQs by describing the rationale, design, implementation, administration, and user feedback of a Web-based graphical food frequency assessment system. Online FFQs are not yet being used widely, and this is due in part to a variety of technical challenges. This section summarizes some of the technical considerations relevant to facilitating wide deployment of online FFQs.

#### Reproducibility

With a view to decreasing completion time and thereby increasing user acceptability, the list of food items in an FFQ normally includes only the most common foods in a region, divided into food groups (fruits, vegetables, etc). As these food lists and their related portion size images vary by location, it is useful to have either a customizable central system or an easily replicable system to help ensure that locally applicable FFQs for different regions can be created easily. Ideally, this system should be inexpensive in order to mitigate financial constraints that could block deployment. Furthermore, any need for technological expertise in customization and administration could hinder reproducibility, so it is important to design these aspects with ease-of-use in mind.

#### Scalability

One of the drivers for developing online dietary assessment methods is the potential to support population-level studies. When operating at this large scale, there is a potential to see high peaks in the system traffic, which are not easily handled. This is an important requirement to be considered in the system architecture.

#### Mobile Friendly

The need to consider deployment on mobile devices and tablets is more and more relevant, considering an increase in the market share of smartphones and tablets as compared with desktops and laptops [24]. The delivery of an FFQ via tablets and smartphones presents particular challenges. For example, due to screen size constraints, it is difficult to present all the portion sizes (usually between 3 and 7 images) on the screen simultaneously. The layout and interaction design has the potential to influence participant responses or increase the task completion time and requires careful consideration.

#### Security

Population studies often store sensitive data, since they usually collect medical information together with personal details. In this scenario, it is important to provide authentication and authorization features, protect the database from unauthorized access, and communicate with the database using a secure protocol.

#### Usability

Empirical data on system usability is important for enabling evidence-based decisions in the design and improvement of further systems. The system should build in the ability to collect metrics such as completion time and usability surveys.

## Methods

### Technical Design

#### Overview

The design of the EatWellQ8 food frequency questionnaire considered the main requirements described in the previous section and assessed and compared these with the main advantages and disadvantages of the currently most-used Web architectures and technologies.

The requirements showed that the system was not intense computationally, pointing to the possibility of using a modern Web architecture called single-page application (SPA) [25]. In this paradigm, all the necessary code (HTML, Cascading Style Sheets, and JavaScript) is retrieved in a single load, and the updates in the view are managed by the code running in the browser. The JavaScript framework for creating SPA proposed by Google is called AngularJS, which is entirely client-side (ie, browser only) [26].

An SPA architecture creates the possibility of using static hosting for delivering the code and media files (eg, food images in this project), which is much cheaper than dynamic hosting (ie, servers) and removes any need for server maintenance.

Beside the static hosting, there were three basic requirements that needed to be fulfilled: user authentication, user authorization, and a secure database. After analyzing several major cloud-computing providers (ie, Amazon Web Services, Google, IBM, and Microsoft), it was clear that the typical Web app architecture could be delivered by any of them. One particular service that stood out during this comparison was Google Firebase for its particular focus in providing the most essential features for developing Web and mobile apps in a very affordable way, which has attracted more than 400,000 developers worldwide. Its main features are a real-time database, user authentication, and static hosting [27].

#### Reproducibility

Since data collection and retention standards are different around the world, a customizable central system may face some practical difficulties for implementation. This was one of the main reasons for choosing to create an easily replicable system using cloud-computing services, which are accessible worldwide.

Data is stored in a JavaScript Object Notation (JSON) document in the Firebase database. In order to facilitate food list modification by nontechnical administrators, the original food table was created as an Excel spreadsheet (Microsoft Corp). The cells were then concatenated (using Excel’s concatenate function) into comma-separated values text, which was then converted to JSON (using a online converter such as convertcsv.com). The JSON was then imported to Firebase. The following object shows a food item structured in JSON, illustrating its human-readable format:

“foods” : [ { “arabic” : “Broccoli in arabic”,“english” : “Broccoli”, “id” : 0 }, ....]}

#### Scalability

Using an SPA approach combined with a Firebase database, all the processing is transferred to the client (browser), which can easily handle simple interactions and functions for rendering the pages. The Firebase Spark Plan (free) can support 100 simultaneous connections with the database (this increases to unlimited simultaneous connections with the Flame Plan which, at the time of writing, costs US $25/month) using a secure https protocol and deliver the pages and images via its global Content Delivery Network [27].

#### Mobile Friendly

In order to design a Web app that can be used readily on mobile devices, the design was based on Bootstrap, a highly popular responsive Web framework. It is open source and has built up a big developer community since its launch in 2011 [28].

The Bootstrap functionalities that played important roles in our implementation were the responsive navigation bar and the modal component; the former creates an adjustable navigation bar that converts into a hamburger icon on small devices, and the latter displays a pop-up window on top of a current page (this was used to be able to display food portion images using the entire screen).

#### Security

Firebase provides a complete authentication feature. Among the possible authentication providers (Facebook, Google account, etc), the email and password combination was enough for this project, although others could also be provided as alternatives. Firebase enables the use of AngularJS combined with its product via the AngularFire library. It provides a 3-way binding between the HTML, the JavaScript, and the database. This means that any modification in one of these parts can be propagated to the other two. For example, a modification of one value in the database triggers an update in the website. This feature becomes even more powerful when different systems are connected to the same real-time database, enabling users to switch between a website and a mobile app, for example, with their data synchronized between the two. Best practices in terms of authentication and page routing are provided by Firebase in the AngularFire Seed, a small open-source project that contains the implementation of the basic features (log in, password reset, data binding, etc) that were used in this project.

Besides the authentication feature, Firebase provides Security Rules for defining authorization. Every time a user authenticates, an internal variable (*auth*) is populated with user information (eg, user unique ID). Using a simple JavaScript-like syntax, authorization was defined in order to prevent unexpected access. The following rules exemplify how to block access (read/write) to new objects and only allow authenticated users to access their own FFQ results:

{“rules”: {“.read”: false, “.write”: false,

“ffq”: {“$user”: {“.read”: “auth.uid === $user”,“.write”: “auth.uid === $user” }}}}

Another important security aspect is communication between the browser and the database. Firebase uses https, which requires encryption in the communication between the browser and Firebase. If a custom domain is desired for the deployment (eg, https://eatwellq8.org), it will be necessary to configure the Domain Name Server according to the records provided by Firebase.

### EatWellQ8 Food Frequency Questionnaire

**Figure 1:**
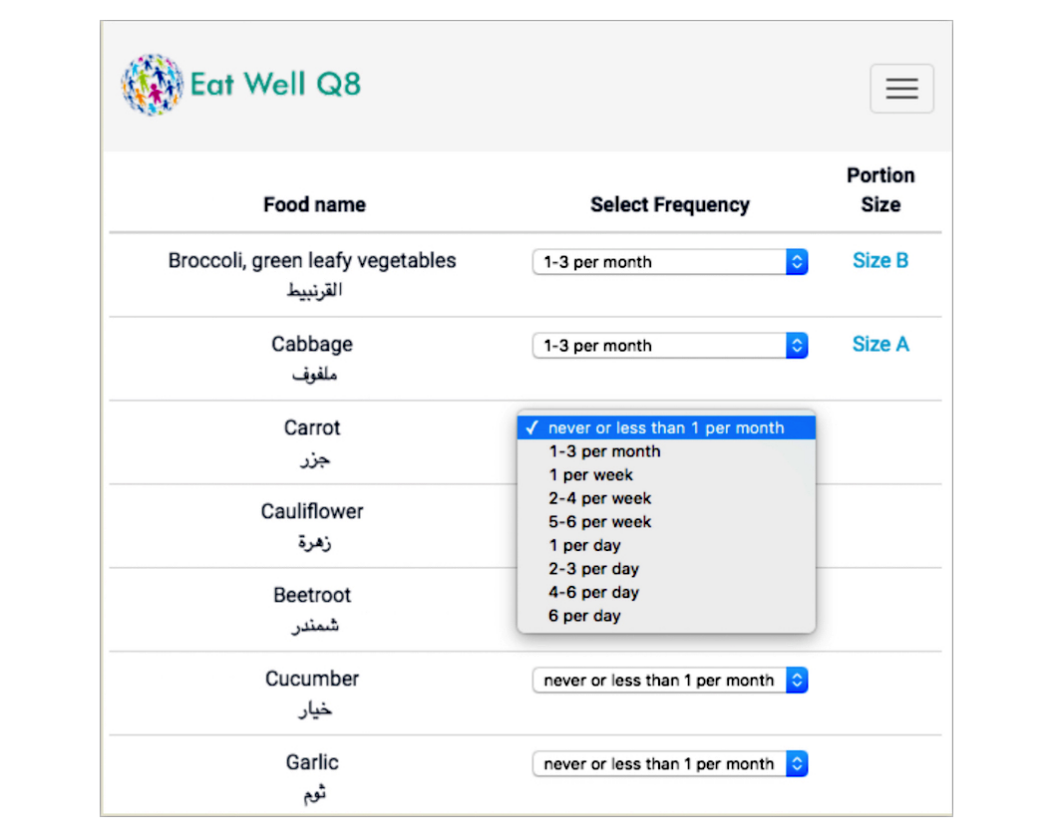
Food items and frequencies presented by the system.

The EatWellQ8 FFQ contains 146 food items (including drinks), adapted from the European Prospective Investigation into Cancer Study [29] and Food4Me FFQs [4] to reflect a Kuwaiti diet. The food names are shown in both English and Arabic. For each item, users indicate consumption frequency during the last month by selecting from 1 of 8 options: “never or less than 1 per month,” “1 to 3 per month,” “2 to 4 per week,” “5 to 6 per week,” “1 per day,” “2 to 3 per day,” “4 to 6 per day” and “more than 6 per day” [4]. Due to the number of options, the selection was implemented via a select element (drop-down list), which is expanded on mobile devices. In order to speed up the completion time, the default choice was set to the first option (“never or less than 1 per month”), so that participants could simply skip an item if they did not consume that specific food item (Figure 1).

**Figure 2:**
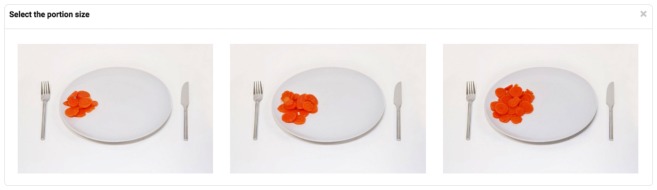
Portion sizes presented by the system.

Users indicated portion size by selecting from 1 of 3 photographs of actual food portion sizes (Figure 2). Other studies have investigated various options to enable users to specify food portion sizes from photographs, including selecting from 1 of 8 portion size photographs [30] and a combination of having 3 portion size photographs to select from combined with 4 radio buttons to indicate portion sizes that were bigger/smaller than those depicted in the photos [31]. For the current system, the decision to present 3 portion size photos was based partly on prior (unpublished, from the FFQ described elsewhere [31]) user data indicating that photos are far more frequently selected than radio button options without an associated photograph and an aim of presenting all the photos to users in an efficient manner even on small screen sizes. Portion size photographs are sometimes labeled using descriptive labels of the portion sizes: for example, small, medium, or large. In our study, the photographs are presented without any labels to avoid potentially biasing the users in their choices. Each time a user selects a food frequency, the appropriate portion images are automatically presented to the user; this is implemented in a pop-up window using the modal component described earlier. After the portion size has been selected, the users’ selections are presented as “Size A,” “Size B,” or “Size C” (see Figure 1).

Although participants are encouraged to complete the FFQ in one sitting/session, it is important to offer the possibility to save the FFQ, in case the user is interrupted or loses Internet connection temporarily. Hence, each food selection is saved individually (after the portion size selection), and the user has the option to retrieve the FFQ of a particular day when returning to the system. A timestamp (format yyyy-mm-dd) is saved together with each FFQ entry in the database, after formatting the JavaScript Date Object, in order to check the existence of an entry for that specific day.

### Usability Metrics

To enable collection of data on system usability and use, the system included a usability survey and also logged usage data. The usability survey was presented after completion of the food frequency questionnaire. A modified version of the System Usability Scale (SUS) [32], originally defined by Brooke [33], was used to assess the user acceptance of the online FFQ. The SUS consists of 10 questions alternating between positive and negative statements, with 5 possible responses from “strongly disagree“ to “strongly agree.” The statements relate to a range of aspects of system use, such as complexity, ease-of-use, and learnability. Each participant’s responses are then scored, providing an overall SUS score between 0 and 100. After this stage, the overall usability of the system is evaluated via a general question, “Overall, I would rate the user-friendliness of this system as,” with the following options: “worst imaginable,” “awful,” “poor,” “fair,” “good,” “excellent,” and “best imaginable.” An additional question (“Have you found difficulties in some part of the system?”) was also presented. In the case of a positive answer, a textual description of the difficulties was requested.

Collecting usage data involved storing browser information and logging user interactions with the system. Details of the browser were collected via the JavaScript Navigator Object. This object is not intrusive, is supported by all major browsers, and contains information such as browser name, platform, version, and language.

In terms of logging user interactions, the system logged timestamps on actions completed during the completion of the FFQ (eg, opening and closing of the portion size selection screen) using the JavaScript Date Object, which contains the time in milliseconds since the beginning of the year 1970 [34]. The timestamps were analyzed for the total time spent completing the FFQ, calculated based on the first and last click interaction with the FFQ. As the system allowed users to stop partway through the FFQ and to return to it within the same day, in order to measure only the periods in which the volunteers were actively engaged in using the system, time intervals greater than 60 seconds without any click interactions were considered interruptions (ie, period of inactivity) and subtracted from the total completion time.

The EatWellQ8 Web-based food frequency questionnaire was deployed in January 2016 as part of a validation study comparing the online FFQ against a preexisting paper version of a Kuwaiti FFQ and a 4-day weighed food record. The study was subject to ethical review according to the procedures specified by the University of Reading Research Ethics Committee (UREC 15/50) and by the Diabetes Institute’s International Scientific Advisory Board and Ethics Review Committee (RA-2015-018) and was given favorable ethical opinions for conduct.

Because the usability study was being performed in parallel with the EatWellQ8 validation study, participant recruitment and eligibility criteria were set by the requirements of the wider study. Participants were recruited in Kuwait as part of the EatWellQ8 study without financial incentive. Recruitment was conducted via the Internet, posters, and social media or word of mouth, mainly from the higher education institutions in Kuwait, during 2016. Volunteers were requested to create an online account on the study website and to complete a screening questionnaire to determine their eligibility to participate in the study. Participants with chronic diseases (eg, diabetes), food allergies or food intolerances, or not within the age range (18-65 years) were not eligible to participate in the study.

**Figure 3:**
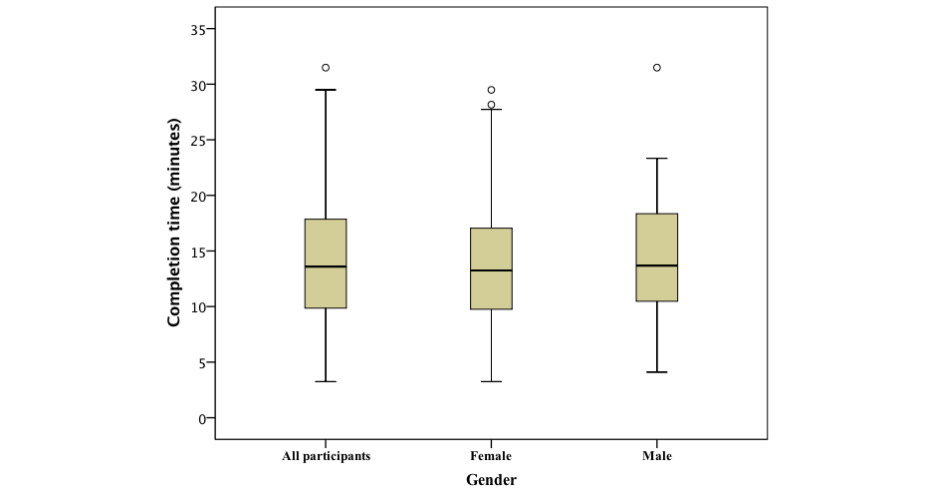
Food frequency questionnaire completion time for all participants (n=163) and by gender (93 female, 49 male).

## Results

A total of 235 participants created accounts in the system, of which 163 completed the FFQ. Of those 163 participants, 142 reported their gender (93 female, 49 male) and 144 reported their date of birth (mean age of 35 years, range from 18-65 years).

Regarding the devices participants used to complete the FFQ, 69 participants used a laptop/desktop computer, 87 used a smartphone, 4 used a tablet, and 3 devices/browsers did not return their JavaScript Navigator Object correctly and hence the device information is not available.

**Figure 4:**
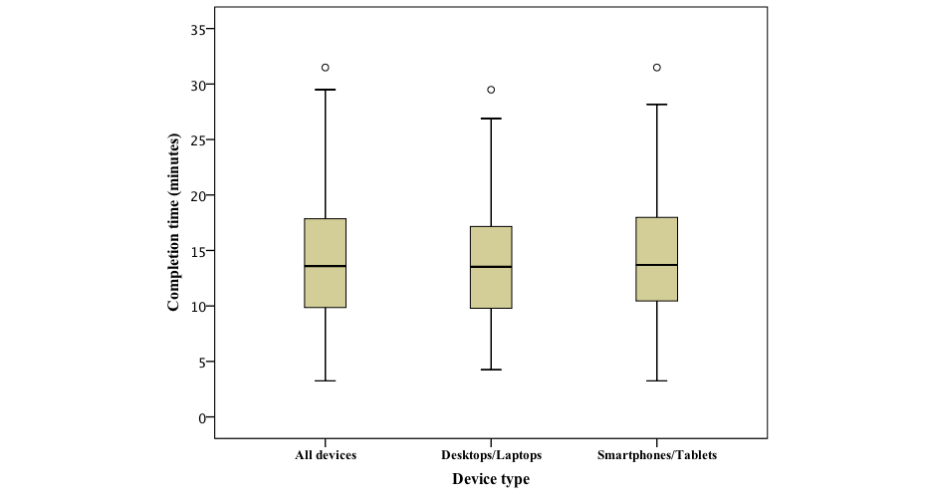
Food frequency questionnaire completion time for all devices (n=163) and by device (69 laptops/desktops, 91 smartphones/tablets).

The mean completion time for all FFQs (n=163), excluding periods of interruption, was 14.2 minutes (95% CI 13.3-15.1 minutes). Female participants (n=93) completed in 14.1 minutes (95% CI 12.9-15.3 minutes) and male participants (n=49) completed in 14.3 minutes (95% CI 12.6-15.9 minutes) (Figure 3). Participants using laptops or desktops (n=69) completed the FFQ in an average of 13.9 minutes (95% CI 12.6-15.1 minutes) and participants using smartphones or tablets (n=91) completed in an average of 14.5 minutes (95% CI 13.2-15.8 minutes) (Figure 4). Out of the 163 FFQs, 71 were completed without any interruptions (ie, there was no gap of more than 60 seconds without any interaction). Considering the 146 food items, the volunteers spent on average 5.84 seconds per food item. As the system collects timestamps just before the portion image presentation (ie, after the frequency selection) and when they are selected (ie, click on the portion image), it was possible to calculate the mean time spent in the portion size selection (4.18 seconds per food item) and by subtraction the rest of the time (1.66 seconds per food item) was considered spent on the frequency selection component of the task. For items where the frequency was “never,” no explicit selection was required.

Regarding the portion size selection, we did not have the timestamp required to separate the time required for image loading from the time required by participants to decide on and select a photo due to the fact that this information cannot be captured by the Web app. However, informal testing with a good Internet connection showed that the pop-up is rendered with the 3 images (around 150 KB in total) in less than 1 second.

**Figure 5:**
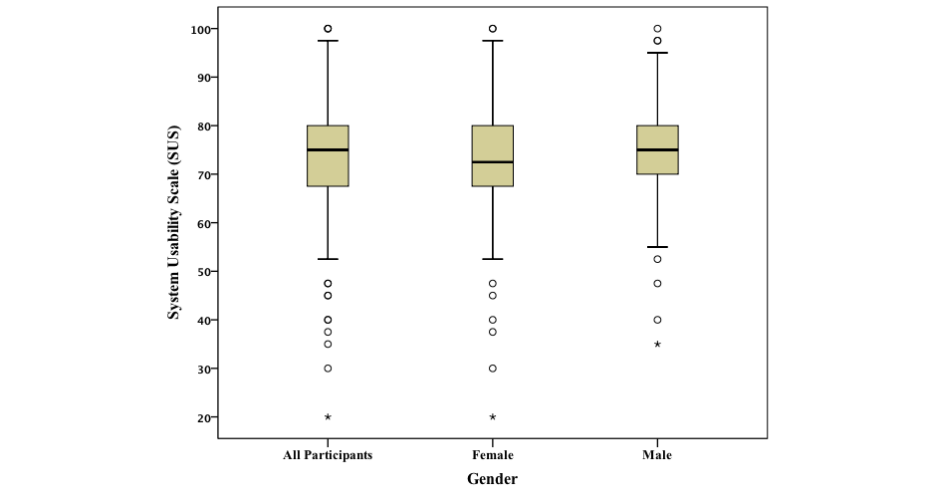
System Usability Scale of the food frequency assessment system by the study participants (n=141) and presented by female (n=80) and male (n=45) for those who reported gender (n=125).

**Figure 6:**
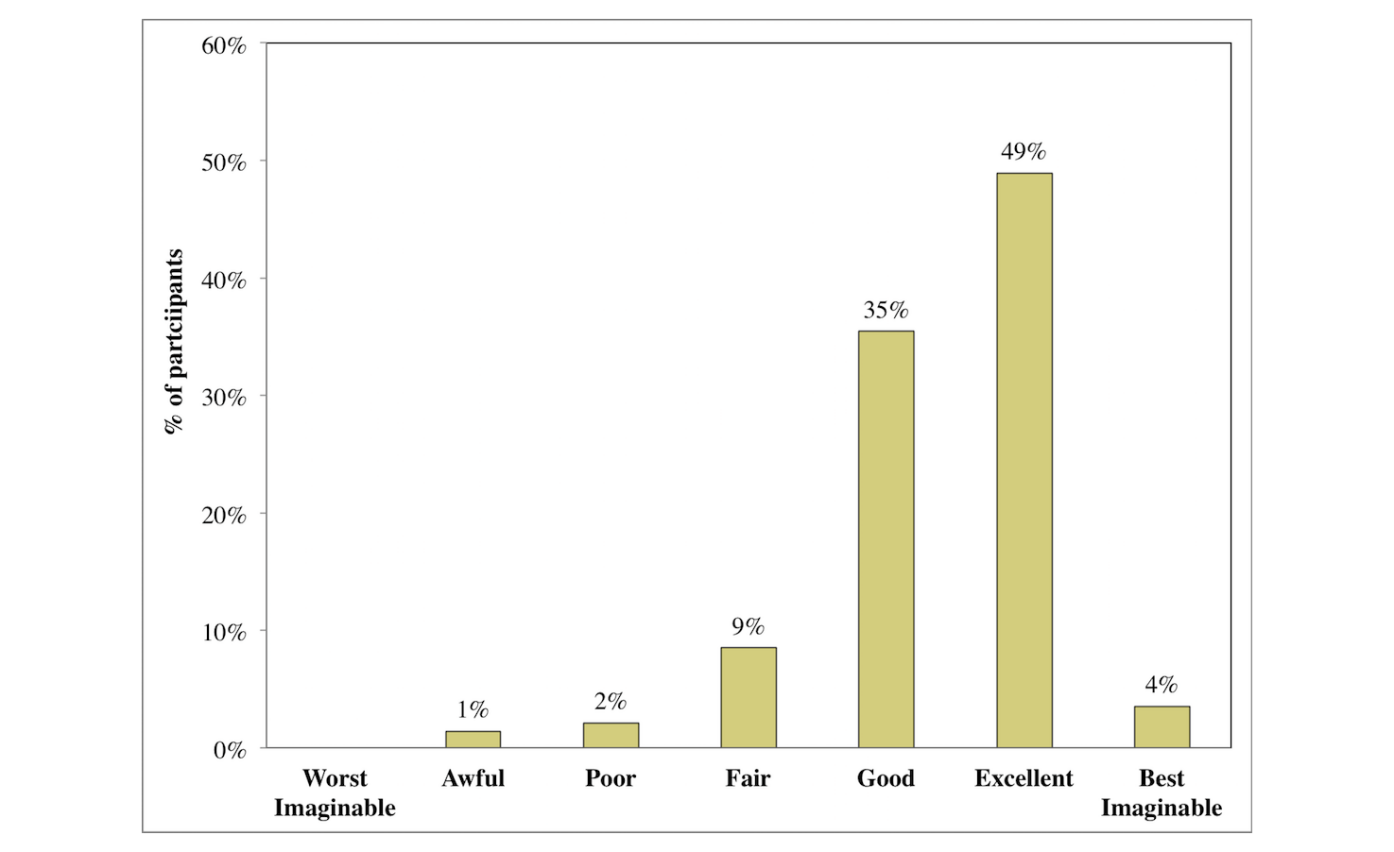
Overall user evaluation of the food frequency assessment system by the study participants (n=141).

For all participants, the usability survey was presented after completion of the FFQ. Of the 141 who elected to complete the usability survey, 125 reported their gender (80 female, 45 male) and 124 reported their date of birth (mean age of 36 years, range from 18-65 years). The median SUS score (n=141) was 75.0 (interquartile range [IQR] 12.5) for all the participants, and of the 125 who reported their gender, the results were 72.5 (IQR 12.5) for female (n=80) and 75 (IQR 11.25) for male (n=45) (Figure 5). Products with a SUS score higher than 70 are considered to be good [35-36]; this is discussed further in the Discussion section. No significant differences were found between genders or age groups (below and above the median) for SUS or completion time. Considering only participants using smartphones or tablets (n=80), the median was 72.5 (IQR 12.5), slightly below the SUS median for desktops and laptops (n=58), which was 75.0 (IQR 12.5). Users’ ratings on the overall user-friendliness of the system (based on the question “Overall, I would rate the user-friendliness of this system as”) were predominantly “good” and “excellent” (Figure 6).

In the final question (“Have you found difficulties in some part of the system?”), 126 volunteers answered “no” and 15 answered “yes.” Further examination of the participants who provided comments (n=13) showed that their responses were more related to the process (eg, “too long and detailed,” “repeated questions,” “gets boring,” and “time consuming”) rather than fundamental problems with the system. Only 3 participants reported fundamental problems and they were related to the portion size pop-up in smartphones. Selected comments related to the usability of the system follow:

The portion size pop-up aspect of the FFQ became a bit tedious. I think it might be slightly more user-friendly if the portion pictures are posted on the website rather than in pop-up form.

The pictures were great and really were on spot with the amounts difference.

It was not clear for me when choosing the portion/size if there was more than a, b, and c. By using mobile it was not easy at all to scroll down the size option.

## Discussion

### Principal Findings

Participants gave the EatWellQ8 system a median SUS score of 75.0 (IQR 12.5). Kurtom and Bangor measured popular services and products and reported a SUS average of 70.14, including Microsoft Excel (54.4), Amazon (79.0), and an automated teller machine (80.5) [35-36]. Products with a SUS score higher than 70 are considered to be good [35]. When using this scale, it is useful to compare results within the same category. A very recent study published the SUS results of an online 24-hour recall system designed and developed during the project myfood24 [37]. For an adult population, it resulted in a SUS median of 68 (IQR 40) for the beta version, and a SUS median of 80 (IQR 25) for the live version. No similar results have been published for online FFQs, but the SUS median from this study indicates good design and user acceptability. We acknowledge potential for selection bias, which could not be quantified. This is further supported by participants’ positive responses relating to the overall quality of the system (Figure 6). We observed similar completion times and SUS medians for completing the FFQ on smartphones/tablets when compared with laptops/desktops, which indicates a good responsive design.

Although retrospective dietary assessment methods such as the FFQ and 24-hour recall require less effort from users than prospective methods using similar technologies (eg, Web-based food diaries), completion times of around 14 minutes for completing the FFQ in full can still be a barrier if participants are not engaged with the study objectives. The challenge of engaging participants to complete data collection could potentially be addressed by providing personalized online feedback, acting as a reward to incentivize participants to complete the FFQ. A newer version of the EatWellQ8 system is currently under development with the ability to provide personalized feedback, which may further improve user satisfaction and interest for investing this amount of time to complete the FFQ.

### Conclusions

We have designed and deployed an online FFQ in a way that encourages reproducibility and is available to be used in other studies, using the same cloud services, for free. In this way, we hope to make a contribution to the wider uptake of digital FFQs and to make more widely accessible their benefits in terms of time and resource savings and suitability to support large-scale studies.

The FFQ we have developed is a responsive website that has been tested on smartphones and tablets using two major mobile operating systems (iOS and Android). It addresses security requirements using features provided by Google Firebase, a cloud-based real-time database service. The user rating of this version from 141 participants was good (75 out of 100, using the SUS), and the completion time calculated from 163 FFQs (14.2 minutes) seems to be acceptable but with room for improvement. This paper is an important landmark in encouraging the research community to publish technical designs and usability information of online dietary assessment methods.

## Abbreviations

EatWellQ8: Eat Well Kuwait
FFQ: food frequency questionnaire
IQR: interquartile range
JSON: JavaScript Object Notation
SPA: single-page application
SUS: System Usability Scale
